# Classification of Skin Disease Using Deep Learning Neural Networks with MobileNet V2 and LSTM

**DOI:** 10.3390/s21082852

**Published:** 2021-04-18

**Authors:** Parvathaneni Naga Srinivasu, Jalluri Gnana SivaSai, Muhammad Fazal Ijaz, Akash Kumar Bhoi, Wonjoon Kim, James Jin Kang

**Affiliations:** 1 Department of Computer Science and Engineering, Gitam Institute of Technology, GITAM Deemed to be University, Rushikonda, Visakhapatnam 530045, India; parvathanenins@gmail.com; 2 Tata Consultancy Services, Gachibowli, Hyderabad 500019, India; siva.jalluri18@gmail.com; 3 Department of Intelligent Mechatronics Engineering, Sejong University, Seoul 05006, Korea; fazal@sejong.ac.kr; 4 Department of Electrical and Electronics Engineering, Sikkim Manipal Institute of Technology, Sikkim Manipal University, Majitar 737136, India; akash.b@smit.smu.edu.in; 5 Division of Future Convergence (HCI Science Major), Dongduk Women’s University, Seoul 02748, Korea; 6 School of Science, Edith Cowan University, Joondalup 6027, Australia

**Keywords:** skin disease, MobileNet V2, Long Short-Term Memory (LSTM), deep learning, neural network, grey-level correlation, mobile platform, Convolutional Neural Network (CNN), MobileNet

## Abstract

Deep learning models are efficient in learning the features that assist in understanding complex patterns precisely. This study proposed a computerized process of classifying skin disease through deep learning based MobileNet V2 and Long Short Term Memory (LSTM). The MobileNet V2 model proved to be efficient with a better accuracy that can work on lightweight computational devices. The proposed model is efficient in maintaining stateful information for precise predictions. A grey-level co-occurrence matrix is used for assessing the progress of diseased growth. The performance has been compared against other state-of-the-art models such as Fine-Tuned Neural Networks (FTNN), Convolutional Neural Network (CNN), Very Deep Convolutional Networks for Large-Scale Image Recognition developed by Visual Geometry Group (VGG), and convolutional neural network architecture that expanded with few changes. The HAM10000 dataset is used and the proposed method has outperformed other methods with more than 85% accuracy. Its robustness in recognizing the affected region much faster with almost 2× lesser computations than the conventional MobileNet model results in minimal computational efforts. Furthermore, a mobile application is designed for instant and proper action. It helps the patient and dermatologists identify the type of disease from the affected region’s image at the initial stage of the skin disease. These findings suggest that the proposed system can help general practitioners efficiently and effectively diagnose skin conditions, thereby reducing further complications and morbidity.

## 1. Introduction

The skin is the largest organ in the human body, consisting of the epidermis, dermis, subcutaneous tissues, blood vessels, lymphatic vessels, nerves, and muscles. Skin can prevent lipid deterioration in the epidermis with liquid such that the skin barrier feature can be improved. Skin diseases can arise because of fungal development over the skin, hidden bacteria, allergic reactions, microbes affecting the skin’s texture, or creating pigment [1]. Skin illnesses are chronic and occasionally may grow into malignant tissues. To minimize their development and proliferation, skin diseases must be treated immediately [2]. Research on procedures to identify the effects of diverse skin diseases based on imaging technology is now mainly in demand. Several skin diseases exhibit symptoms that might take considerable effort to treat such patients as they grow for months before they are diagnosed. Prior work in dermatological computer-aided classification has lacked medical experts’ generalization capability due to insufficient data and a focus on standardized tasks such as dermoscopy that refers to the examination of the skin using skin surface microscopy. It is possible to efficiently and reliably classify skin diseases through computer-aided diagnosis to prescribe medication based on patients’ symptoms [3]. This work presents a robust mechanism that can accurately identify skin diseases through supervisory approaches that lower diagnosis costs. A grey-level co-occurrence matrix is used for assessing the progress of diseased growth. The diagnosis’s accuracy is significant in a comprehensive assessment of the abnormality for better treatment and reduces medication costs.

The inclination of skin diseases shows a multiplicity of forms, lack and misdistribution of qualified dermatologists, and the need for timely and accurate diagnosis calls for data-driven diagnosis. The advancement of lasers and photonics-based medical technology has made it possible to diagnose skin diseases much more quickly and accurately. However, the cost of such diagnosis is still limited and expensive. Deep learning models [4,5,6,7] are comparatively efficient in performing the classification process from the images and the data. There has been a demand in the field of healthcare diagnosis in precise identification of the abnormality and classifying the category of the disease from the X-ray, Magnetic Resonance Imaging (MRI), Computer Tomography (CT), Positron Emission Tomography (PET) images, and the signal data like the Electrocardiogram (ECG), Electroencephalogram (EEG), and Electromyography(EMG) [8,9,10,11,12,13,14]. The precise identification of the disease category will assist in providing better treatment for patients. Deep learning models can solve critical problems by automatically identifying the input data features, and the deep learning models are adaptable to the change in the considered problem. Deep learning models will acquire the inferred data to identify and explore the features in the unexposed data patterns with even low computational models resulting in considerable efficiency. That has motivated the authors in considering a deep learning model in classifying the skin disease category from the affected region’s image proposed work.

This study used a dataset consisting of seven skin diseases: Melanocytic nevi, Benign keratosis-like lesions, Dermatofibroma, Vascular lesions, Actinic keratoses, Intraepithelial carcinoma, Basal cell carcinoma, and Melanoma. This dataset contains more than 10,000 dermatoscopic images. A random (rand) function is applied to split the data into the training data (7224) and validation data (1255). The considered dataset is slightly imbalanced because some skin diseases are more, and some are less in number. To overcome such problems, we used data augmentation, and this technique balances the data and generates more images either by rotations or transformations from the existing data.

The main objective of the article is to bring in the state of art technique, namely the MobileNet V2 [15] with LSTM [16] component for the purpose of the precise classification of skin disease from the image that is captured from the mobile device. The practical implication of the model is to design the app through which the image of the affected region of the skin is captured to determine the class of the skin disease. The MobileNet V2 model is computationally efficient to work with light-weight computational devices and working with low resolution images has motivated the authors to choose the MobileNet V2 model and LSTM is efficient in handling the gradient disappearing issue over the iterations in the neural networks that assist in faster training of the model [17,18]. The proposed model would assist medical practitioners and the patient in an effective non-invasive way of diagnosis of the disease with least possible cost and workforce.

The rest of the article is organized as follows. Section 2 describes the related work in detail on recent technologies for recognizing skin disease. Section 3 is about the proposed approach through Fuzzy Recurrent Neural Networks to classify the type of skin disease. Section 4 describes results and discussion, followed by a conclusion and future work in Section 5.

## 2. Related Work

Several existing approaches are mechanized to recognize and classify skin diseases. Most of the diagnosing methods rely on imaging technology, and the epidermal recognition of such skin diseases does not need radiological imaging technologies. They can recognize the condition based on the standard images through image processing techniques, including image transformation, equalization, enhancement, edge detection, and segmentation [19,20,21]. The skin images that are captured for disease identification and classification are processed and fed as input for the advanced artificial intelligence approaches like Machine Learning, Deep Learning, Artificial Neural Network, Convolutional Neural Network, Back Propagation Neural Network, and classifiers such as Support Vector Machines, Bayesian classifier for the prediction of the type of skin disease.

Skin diseases are also classified through the necessary image processing approaches like morphological operations for skin detection [22,23]. Morphological opening, closing, dilation, and erosion mostly rely on the binary image generated through the thresholding, and resultantly at most care must be taken to determine the optimal threshold value. The morphological-based operations may not be suitable in estimating the damaged region’s growth based on the image’s texture. Genetic Algorithm (GA) established an approach for skin disease classification [24,25]. The Genetic Algorithm does have challenges like too much time to converge towards the solution [26]. The model never grants the global best solution which would not result in a reasonable outcome [27].

Alam et al. [28] automated the detection of eczema using image processing through a support vector machine which involves various phases that include segmentation of the acquired image, followed by feature selection using texture-based information for more accurate predictions, and finally making use of the Support Vector Machine (SVM) for evaluating the progress of eczema as presented by I. Immagulate [29]. The Support Vector Machine model is not appropriate to handle the noisy image data [30]; identifying the feature-based parameters is significant when working with SVM. It will underperform if the number of parameters at each feature vector is more significant than the number of training data samples.

Artificial Neural Networks (ANN) [31] and Convolutional Neural Networks (CNN) [32] are the most predominantly used techniques in identifying and diagnosing anomalies from radiological imaging technologies. Skin diseases diagnosis using the CNN approach showed that the results are promising [33]. Yet, the CNN models are not scaled and rotation invariant which is a challenging task to work with images captured using a mobile device or a digital camera. The ANN-based model for earlier detection of breast cancer is through image processing; either of the neural network approaches methods need tremendous training data for the model’s considerable performance which requires a lot of computational effort [34]. The neural network models are more abstract, and we do not have the accessibility to customize the model. Moreover, in ANN, with the increase in image resolution, the number of trainable parameters increases significantly which results in tremendous efforts for training. The ANN model suffers with diminishing and exploding the gradient. CNN does not interpret the object’s magnitude and size in its observations [35].

The Fine-Tuned Neural Network-based [36] skin disease classification model has achieved a reasonable accuracy of 89.90% for the validation set. However, it needs a significant effort to calibrate the network components to attain the desired accuracy. Back Propagation Neural Network [37] is a supervisory learning model that works on the gradient descent principle that refines the weights based on the error rate. However, the model fails to work with noisy data. The other primary concern is that when the elements are fed with new weights, it forgets the previously associated weight, leading to a considerable impact on the previous associations [38]. Fuzzy Recurrent Neural Networks (FRNN) [39] and Takagi–Sugeno–Kang Fuzzy Classifier [40] have attained a reasonable accuracy for divergent classification problems, and they perform exceptionally better for handling a variable size input without impacting the model. Recurrent Neural Network (RNN) can process the data with the available arbitrary memory, unlike most of the neural network models that need an auxiliary memory for processing. However, RNN is comparatively slow due to heavy computational needs, and FRNN requires a tremendous effort in classifying the patterns from the image data and consumes noticeable computational time [6].

The image is classified based on intensity though a statistical approach, namely Gray Level Co-occurrence Matrix (GLCM) extracts the features that appear in the acquired image, usually the textured-based parameters [41]. GLCM determines the instance amplitude tabulation concerning a particular combination of attributes of intensity values in an image. However, GLCM needs considerable computational efforts, and characteristics are not invariant with rotation and texture changes [42].

Bayesian classification is among the approaches used in skin disease classification [43]. The approach is used in the classification of the image among the various trained disease image datasets. Still, the Naïve Bayes classification fails in independent predictors; the zero-probability problem makes it challenging to implement in the multi-objective-based domain. The Naïve Bayes classifiers are not suitable to handle unsupervised data classification [44]. The Decision Tree [45] algorithm is a widely used approach for skin disease classification, prediction of lower limbs ulcers and cervical cancer. The Decision Tree model needs a tremendous amount of training and a considerable accuracy level. A small change in the input data would result in an exponential change in the outcome and make the model insatiable. Additionally, the model needs comparatively more memory, and resultantly the Decision Tree model needs more computational time [46].

K-Nearest Neighbor (KNN) [47] is the predominantly used classification model widely used in forecasting and predictive models. The models do not need training of the model. Moreover, the accuracy of the KNN model is considerably high [48]. The KNN models are not appropriate to use with larger-size data models, as it may take a significant time in performing the predictions of the outcome. In addition, the model performs poorly when working with high dimensional data with inappropriate feature information, which might impact the performance of the model in accurate predictions [49], which has made the model inappropriate for the skin disease classification.

Skin disease classification through the ensemble models [50] yields higher accurate outcomes by combining multiple prediction models. Ensemble models have an overfitting issue, and the ensemble model fails to work with unknown discrepancies between the considered sample and population [51,52]. Deep Neural Network model-based skin disease classification [53,54] has exhibited a notable performance in classifying skin diseases. Still, the experimental studies have shown that the model is not suitable for multi-lesion images. Deep Neural Network models need a considerable training level to attain a reasonable accuracy that requires more computational time.

Cross correlation-based model for classification of the feature extraction [55], where both the spatial and the frequency features are considered for feature selection using visual coherency. The cross-correlation models are robust against the background fluctuations. Resultantly, the predictions are more accurate. Additionally, working in the frequency domain needs considerable effort in creating the experimental setup and obtaining the results.

The proposed model is associated with the mobile application, and there are many other such experimental applications designed for the ease of assessment of the diseases. Lee, H.Y. et al. [56] presented the influence of text messaging on the benefits of human papillomavirus (HPV) vaccination and noticed a sharp rise in HPV vaccine consumption in targeted communities. In another study proposed by Weaver et al. [57] to address screening intake, cancer screening services have also used text messages. Ijaz et al. [58] proposed a model on IoT for healthcare for patients to access remotely and utilize the healthcare gadgets to analyze and monitor their health through bio-medical signals and intimately model the healthcare professionals in case of an emergency. Table 1 summarizes the various machine and deep learning approaches for image classification.

## 3. Methodology

In this section, integrating the LSTM with the MobileNet V2 is explained with an architecture diagram. MobileNet V2 is used in classifying the type of skin disease, and LSTM is used to enhance the performance of the model by maintaining the state information of the features that it comes across in the previous generation of the image classification.

### 3.1. MobileNet Architecture Model for Image Classification

As opposed to MobileNet V2 [63], MobileNet [4] is a CNN-based model that is extensively used to classify images. The main advantage of using the MobileNet architecture is that the model needs comparatively less computational effort than the conventional CNN model that makes it suitable for working over mobile devices and the computers that work over lower computational capabilities [64,65,66]. The MobileNet model is a simplified structure that incorporates a convolution layer that can be used in distinguishing the detail that relies on two manageable features that switch among the parameter’s accuracy and latency effectively. The MobileNet model is advantageous in reducing the network size [67].

MobileNet [68] architecture is equally efficient with a minimum number of features, such as Palmprint Recognition [17]. The architecture of MobileNet is depth-wise [69]. The fundamental structure is based on different abstraction layers, a component of different convolutions that appear to be the quantized configuration that assesses a regular problem complexity in-depth. The complexity of 1 × 1 is called point-wise complexity. Platforms to make in-depth are designed to have abstraction layers with structures in-depth and point through a standard, rectified linear unit (ReLU). The resolution multiplier variable ω is added to minimize the dimensionality of the input image and each layer’s internal representation with the same variable.

The feature vector map of size $F_{m}\times F_{m}$ and the filter is of size $F_{s}\times F_{s}$ the input variable is denoted by p, and the output variable is recognized as q. For the core abstract layers of the architecture, the overall computation efforts are represented by the variable $c_{e}$ and may be assessed through the following Equation (1):

(1) $$c_{e}=F_{s}\cdot \;F_{s}\cdot \omega \cdot \alpha F_{m}\cdot \;\alpha F_{m}+\omega \cdot \rho \cdot \alpha F_{m}\cdot \;\alpha F_{m}$$

The multiplier value is context-specific, and for the experimental analysis in skin disease classification, the value of multiplier ω is considered to be in the range 1 to *n*. The value of the variable resolution multiplier identified by α is deemed to be 1. The computational efforts are recognized through the variable $\;cost_{e}$ can be assessed through Equation (2) stated below:

(2) $$cost_{e}=F_{s}\cdot F_{s}\cdot \omega \cdot \rho \cdot F_{m}\cdot \;F_{m}$$

The proposed model incorporates the depth-wise, and point-wise convolutions are bounded by the depletion variable identified by the variable d that is approximated through the Equation (3) stated below:

(3) $$d=\frac{F_{s}\cdot F_{s}\cdot \omega \cdot \alpha F_{m}\cdot \alpha F_{m}+\omega \cdot \rho \cdot \alpha F_{m}\cdot \alpha F_{m}}{F_{s}\cdot F_{s}\cdot \omega \cdot \rho \cdot F_{m}\cdot F_{m}}\;$$

The two hyper-features width multiplier and the resolution multiplier help adjust the optimal size window for accurate prediction based on the context [70]. In the proposed model, the input size of the image is 224 × 224 × 3. The first two values (224 × 224) indicate the height and width of the image. These values should always be greater than 32. The third value suggest that it has 3 input channels. The proposed architecture has 32 filters, and the filter size is 3 × 3 × 3 × 32 [71].

**Figure 1:**
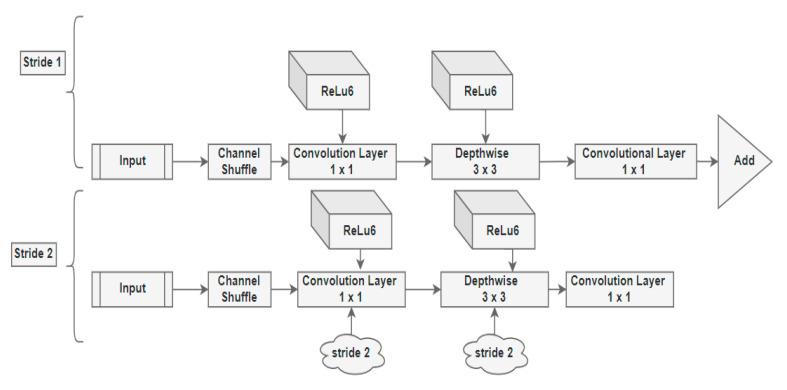
The architecture of MobileNet V2 model.

The principle underneath the MobileNet architectures is to substitute complicated convolutional layers in which each layer comprises a convolutionary layer of size 3 × 3 that buffers the input data, accompanied by a convolutional layer of size 1 × 1 pointwise that incorporates these filtered parameters to build a new component as shown in Figure 1. The concept mentioned above is to simplify the model and make it faster than the ordinary convolutional model.

### 3.2. Design Model MobileNet

The MobileNet V2 architecture comprises the residual layer with a stride of 1 and the downsizing layer with a stride of 2 alongside the ReLu component. The architecture of the same is represented in Figure 1.

Both residual and downsizing layer encompass 3 sub-layers each.

- The 1 × 1 convolution with the ReLu6 is the first layer.
- Depth-Wise Convolution is the second layer in the architecture. The Depth-Wise layer adds a single convolutional layer that performs a lightweight filtering process.
- 1 × 1 convolution layer without non-linearity is the third layer in the proposed architecture. In the third layer, the ReLu6 component is used in the output domain.
- ReLu6 is used to ensure the robustness used in low-precision situations and improvise the randomness of the model.
- All the layers have the same quantity of output channels within that overall sequence.
- The filter of size 3 × 3 is common for contemporary architecture models, and dropout and batch normalization are used during the training phase.
- There is a residual component to support the gradient flow across the network through batch processing and ReLu6 as the activation component.

**Figure 2:**
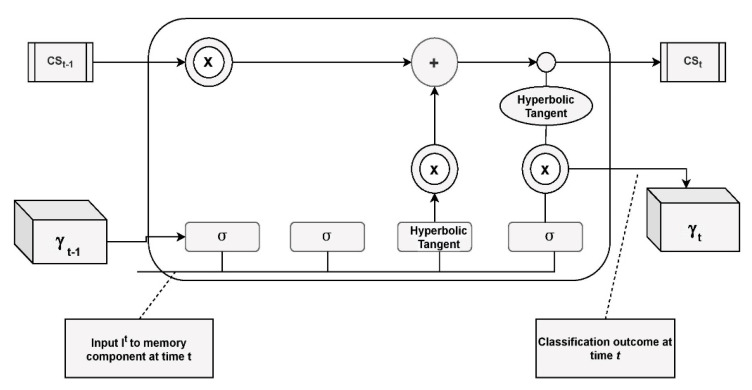
Architecture of LSTM component.

In Figure 2, the symbol σ represents the sigmoid layer, Hyperbolic tangent (*tanh*) is the layer for the non-linearity layer. $cs_{t-1}$ designates the current cell state, and $cs_{t}$ is in concern to the next cell state. $\gamma_{t-1}$ designates the present hidden component and $\gamma_{t\;}$ represents the next hidden state. X designates the scaling of the data, and the symbol + is for summation of the data.

### 3.3. MobileNet V2 with LSTM

LSTM [16] is the component that is extensively used with recurrent neural network architectures. It is capable of reliance on its learning sequence on pattern estimation problems. Memory blocks are managed by memory cells that comprise an input and outlet gate, a forgotten gate, and a window connection encompassed in the abstract LSTM layer module. The calculations describe the activation function for the persistent abstract LSTM memory module. The LSTM module encompasses memory. The state is interpreted as $P_{t}$ at the time t over the hidden state vector $v_{t}$ of the input:

(4) $$\mathrm{Input}\;\mathrm{Gate}:\;\alpha_{t}=\sigma \left(i^{t}W_{i\alpha }+\gamma_{t-1}W_{\gamma \alpha }+cs_{t-1}W_{cs\alpha }+\alpha_{bias}\right)\;$$

(5) $$\mathrm{Output}\;\mathrm{Gate}:\;\beta_{t}=\sigma \left(i^{t}W_{i\beta }+\gamma_{t-1}W_{\gamma \beta }+cs_{t}W_{cs\beta }+\beta_{bias}\right)$$

(6) $$\mathrm{Forget}\;\mathrm{Gate}:\;f_{t}=\sigma (i^{t}W_{if}+\gamma_{t-1}W_{\gamma f}+cs_{t}W_{csf}+f_{bias})$$

(7) $$\mathrm{Cell}\;\mathrm{State}\;\mathrm{Gate}:\;cs_{t}=f_{t}\;\cdot cs_{t-1}+\alpha_{t}\cdot \;\tan\gamma \left(i^{t}W_{ics}+\gamma_{t-1}W_{\gamma cs}+cs_{bias}\right)$$

(8) $$\mathrm{LSTM}\;\mathrm{outcome}:\;\gamma_{t}=\beta_{t\;}\cdot \;\tan\gamma \left(\;cs_{t-1}\right)$$

**Figure 3:**
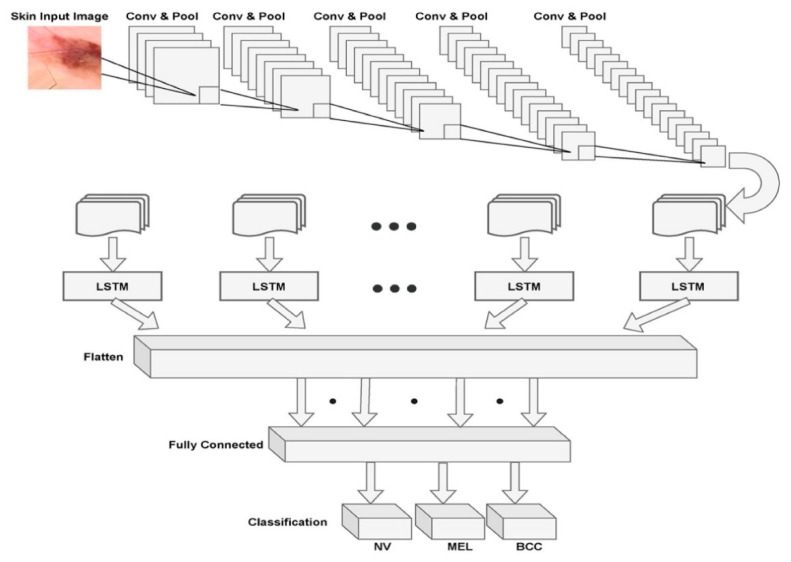
Architecture of the proposed model with MobileNet V2 and LSTM.

From Equations (4)–(8), the variable $\;i^{t}$ is the input to the LSTM block at the time ‘*t*’. The weights $W_{i\alpha },\;\;W_{i\beta }$, $W_{if}$, $W_{ics}$ are associated with input gate, output gate, forget gate, and cell stated gate, respectively. $W_{\gamma \alpha },W_{\gamma \beta },W_{\gamma f}$ are the weights associated with the hidden recurrent layer. The integration model is shown in Figure 3.

Figure 3 presents the overall architecture of the MobileNet V2 with the LSTM model with a combination of set of convolutions and max pooling layers and the LSTM component that is attached to the flattening layer of the model. The fully connected layer that performs the correlation of the identified features with the pre-existing data through training. Finally, the softmax layer that determines the probabilities of various classes of diseases.

### 3.4. Grey-Level Correlation Matrix

One strategy of texture attribute extraction is the Grey-Level Co-occurrence Matrix (GLCM) [72] approach with the localized intensity coefficient’s recurring sequence. GLCM gives the spatial distribution structure of the color and intensity of the pixel, which is determined by the distribution of intensity levels within the window. GLCM focuses on intensity histogram tabulation for a mutation of various pixel intensity values in an image. The association among the two pixels i.e., reference and neighbor pixel through GLCM model using the Equation (9). The variable *Om* designates the occurrence matrix of dimension m × m, where m represents the image’s grey levels:

(9) $$O_{m}\left[i,j\right]=p_{ij}$$

In the Equation (9) stated above, the variable $m_{ij}$ denotes the histogram of the intensity value (i,j) at the dimension m of the image. The components of the occurrence matrix are normalized through Equation (10):

(10) $$M(ij)=\frac{O\left[i,j\right]}{\sum_{i=0}^{m-1}\sum_{j=0}^{m-1}O\left[i,j\right]}$$

By normalization, matrix components have a dimension scale from 0 to 1 that can be modified as a function of likelihood. The variable (k,m) represents the number of elements dimensions of the feature vector that is a set of number of elements and the dimensions, the feature vector can be assessed through Equation (11):

(11) $$fv\left(k,m\right)=\overset{m-1}{\underset{i=0}{\sum }}\overset{m-1}{\underset{j=0}{\sum }}{\left(i-j\right)}^{2}M\left[i,j\right]$$

The GLCM approach is used in approximating the disease growth based on the obtained texture-based information. The GLCM is used in evaluating the skin disease of the proposed model.

### 3.5. Implementation Platform

This experiment was performed on an online compiler named Kaggle [73] with an Intel core ™i7-8550U CPU @ 1.99 GHz accelerated by RADEON (TM) 530 Graphics 8 Gb memory. In the implementation process, on training with the model with a tremendous amount of data for better accuracy, the ordinary CPU might take considerable execution time. To overcome that, a GPU accelerator is used to build the model to save a large amount of time. The in-depth learning approach, represented in our paper, is built using the PyTorch Deep Learning framework [74].

### 3.6. Libraries

The libraries used in our model are NumPy, pandas, os, matplotlib. pyplot, shutil, seaborn, and torchvision as stated by Declan V. [75]. The Matplotlib, pyplot, and Seaborn libraries are used for image operations and plotting, such as graphs, charts, and tables. The Shutil and os libraries offer path and directory operations on files and the collection of files. For model building such as classification report, ROC curve, and confusion matrix, we import the torchvision and seaborn libraries. The numpy and pandas are the most popularly used libraries for array processing and data analysis (series and data frames).

### 3.7. Dataset Description

The dataset plays a crucial role in the training of our proposed neural networks for automated diagnosis. The dataset named HAM10000 is the skin disease dataset that has been extracted from the Kaggle, which has served as a benchmark database downloaded from the source [76]. The dataset comes in metadata format such as comma-separated values file (.CSV), consisting of age, gender, and cell type. This dataset contains more than 10,000 dermatoscopic images that are collected from different people around the world. The dataset also provides additional tips and tricks to overcome certain challenges such as overfitting and limited data, which will help in increasing the model’s accuracy and performance. In this dataset, we have seven different types of skin problems in our dataset, namely Melanocytic Nevi (NV), Benign Keratosis-like Lesions (BKL), Dermatofibroma (DF), Vascular Lesions (VASC), Actinic Keratoses, and Intraepithelial Carcinoma (AKIEC), Basal Cell Carcinoma (BCC), and Melanoma (MEL). There is an imbalance in the number of skin images in each type of lesion present in the dataset. To avoid this imbalance, we performed data augmentation techniques to balance all types of lesions to the same range of images. The dataset is divided into three parts: training data, validation data, and testing data of 85%, 5%, and 10%, respectively, to enhance our model’s generalization. The model is evaluated against the ground facts that are associated with the training dataset. The target size of the images for our proposed model is 224 × 224. This research aims to determine the accuracy in diagnosing skin cancer on dermatoscopic images using our proposed approach.

## 4. Results and Discussion

In this section, the results of the proposed model are discussed in detail. The proposed MobileNet V2 with LSTM performance is evaluated through the hyperparameters like training and validation loss measures that determine the proposed model’s capabilities. The proposed model’s learning rate at various training levels is discussed in the current section. The performance evaluation with other existing approaches in terms of Sensitivity, Specificity, Accuracy, Jaccard Similarity Index (JSI), and Mathew Coefficient Correlation (MCC) are presented. The proposed model’s computational time is evaluated as a part of performance evaluation and compared against the existing approaches on performing the classification over similar data.

### 4.1. Performance Evaluation of Proposed Model

The experiment is carried out on the dataset discussed in Section 3. The proposed model’s results on implementation and the statistical analysis through various performance evolution metrics that include, but are not limited to, accuracy measures determine how many times the proposed MobileNet V2 model with the LSTM model is successfully classifying the skin disease.

To make a reasonable contrast among various approaches concerning the implementation configurations, the authors decided to standardize pivotal parameters throughout all the studies. Table 2 represents the parameters that are considered in the implementation of the proposed model.

**Figure 4:**
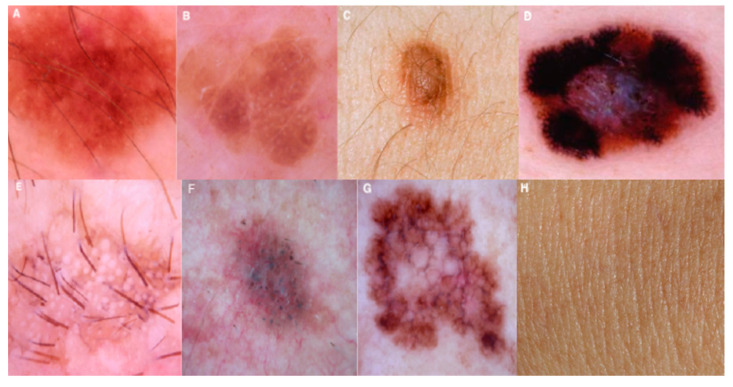
Images of various image classes from HAM10000 dataset. The image of various diseases are as follows (**A**) Melanocytic Nevi, (**B**) Benign Keratosis-like Lesions, (**C**) Dermatofibroma, (**D**) Vascular Lesions, (**E**) Actinic Keratoses and Intraepithelial Carcinoma, (**F**) Basal Cell Carcinoma, (**G**) Melanoma, and (**H**) Normal skin image are presented.

**Figure 5:**
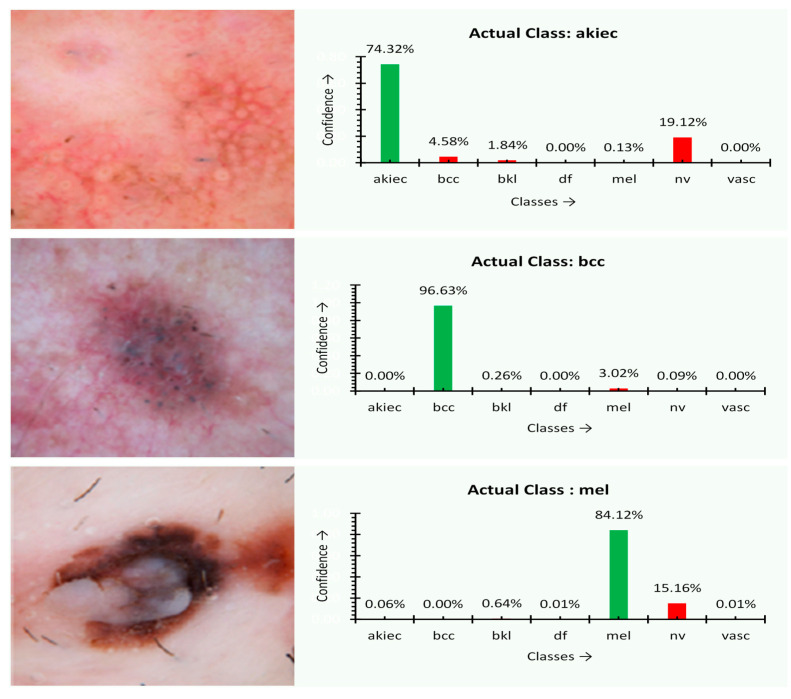
Classification confidence and resultant output images with regular training.

At first, the experiment was performed over several images, and the type of disease is assessed through the proposed MobileNet V2 with the LSTM approach. The outcome of the experiment is shown in Figure 4. The charts next to the skin images in Figure 5 of the experimental outcome represent the percentage of confidence that the disease was observed in the corresponding images of a particular class of disease trained previously. The actual type of disease based on the actual ground facts is also presented. For akiec, bcc, and mel classes, the result appears to be precise. The predicted confidence is on par with the ground reality. The akiec class holds the confidence of 74.32%, 55.2% more than the peer classes. On the other hand, both the mel and bcc class instances are ideally classified with 84.12% and 96.63% confidence, respectively.

**Figure 6:**
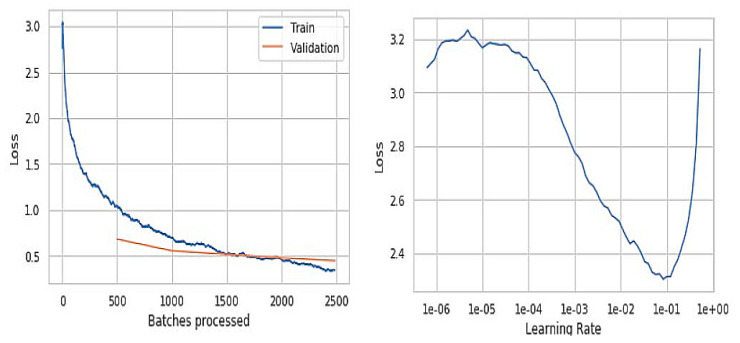
Resultant outcomes post optimizing the training rate.

**Figure 7:**
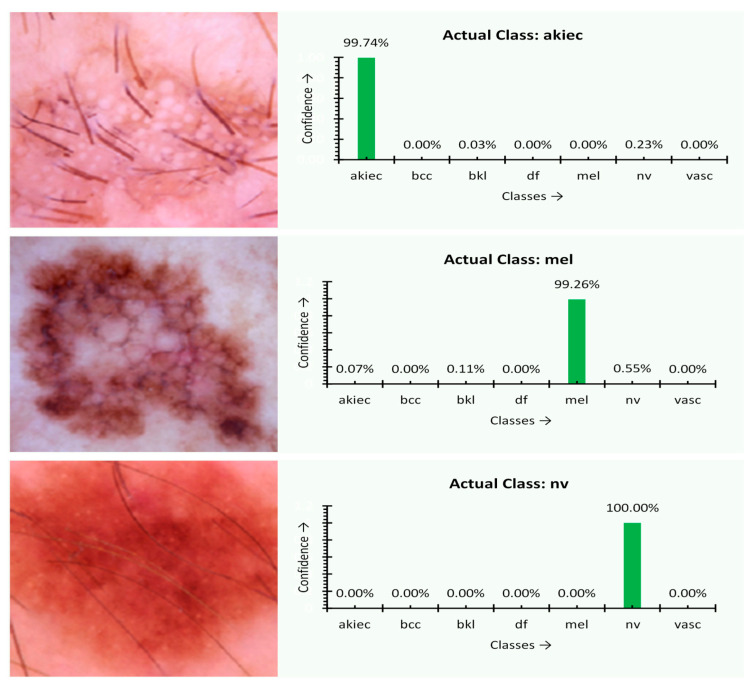
Classification confidence and resultant output images of the final model.

The graphs represented in Figure 6 are obtained from the initial trained model, where the training model loss is better than the validation loss. The left graph indicates the number of batches processed versus loss obtained during the training and the validation phases. The batch size value in the initial model is 100, which is used to speed up the training data. The training and validation loss alongside the learning-rate is presented in Figure 7, and they are significant in determining the overfitting and underfitting of the proposed model. When the validation loss is ahead of the training loss, the model may end up overfitting, and when they are almost equal, it would be an under-fitting problem.

The fact we observed is that the accuracy in predicting the input skin images is slightly distorted. The right graph represents the learning rate versus loss obtained. This non-linear graph resulted in lower values at specific points, challenging, leading to higher epochs and increasing the time complexity.

Figure 6 with graphs and outputs is observed from the trained model before improvements of the training data, and Figure 7 presents the results that were obtained from the trained model after the slight improvements in terms of epochs, batch size, and data augmentation values. The batch size is reduced from 100 to 50 in order to reduce the computational time and also overcome the lower generalization results and higher loss values. The epochs value was increased by 20 to gain more accuracy. The data augmentation is also performed to reduce the over fitting while training and minimizing the error rate. The batch size is kept more for speedup of the previous model’s training data, which ended up getting lower generalization results. The graph represents the loss values versus batches processed in which we got higher loss values compared to the improvised model. Even the learning rate of the previous model is comparatively low when compared to the final model. The learning rate is the hyper-parameter that determines the weight of the network component. If the learning rate is too low, it becomes a challenging task and can also lead the process to get stuck.

**Figure 8:**
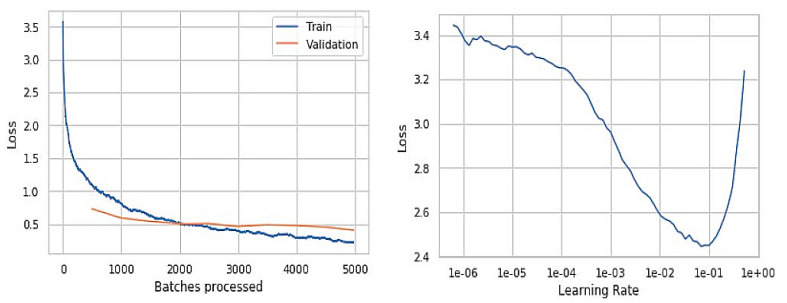
The training, validation, and learning rate of the final model.

To overcome the drawbacks mentioned above, we reduced the batch size to a much smaller size to have faster convergence, resulting in better-optimized results. We increased the learning rate, which resulted in getting better outputs at training fewer epochs. Figure 8 represents the training and validation loss of the batch processing alongside the model’s learning rate upon improvising the model’s training. It can be observed from the graphs that the model has improvised performance at a considerable level. The proposed model’s value is assessed through various performance evaluation metrics like Sensitivity, Specificity, Accuracy, JSI, and the MCC. The models mentioned above’ value is assessed through the True Positive, True Negative, False Positive, and False Negative values assessed through the repeated experimentation of the proposed approach. The True Positive value is about precise identification of the region of disease; True Negative represent the preciseness of the non-disease region of the disease that is evaluated from the image captured. The False Positive represents the number of times the proposed approach fails in recognizing the class of disease accurately, and False Negative determines the number of times the proposed model misinterprets the non-disease region as the disease region.

The Figure 6 and Figure 8 are the resultant hyperparameter graphs obtained on the execution of the proposed model. In either of the graphs, it can be observed that the training and the validation loss curves are close to each other, which depicts an optimal classification of the skin disease. The learning curve presents the reasonable level of learning aspect of the model.

### 4.2. Comparison with Past Studies

The values are evaluated on repeated execution of the proposed model with a varied training level. The performance of the proposed model is compared against a Heuristic Approach for Real-Time Image Segmentation (HARIS) [25], a Fine-Tuned Neural Networks (FTNN) approach [77], a Convolutional Neural Network (CNN) [32], the VGG-19 model [78], and MobileNet models [72,79].

In evaluating the proposed model’s performance, the experimentation is repeatedly executed over the auxiliary computer on repeated execution of the model. The evaluations are done in concern to the number of times the proposed model accurately classifies the skin disorder that is considered the True Positive and correctly identifies that the image is not of that particular skin category as True Negative. The number of times the proposed model recognizes the disease correctly is considered the False Positive. The number of times the proposed model misinterprets the skin disease is assumed as the False Negative. The approximated values of the True Positive, True Negative, False Positive, and False Negative are considered in evaluating the metrics like Sensitivity, Specificity, and the Accuracy of the proposed model.

The values of the various evaluation metrics like Sensitivity (Sen), Specificity (Sep), Accuracy(Acc), Jaccard Similarity Index (JSI), and Matthews Correlation Coefficient (MCC) are presented through the Equations (12)–(16) with respect to the obtained True Positive, True Negative, False Positive, and False Negative values on experimentation. The metrics determines the preciseness of the model in correctly classifying the class of the skin disease.

(12) $$Sen=\frac{True_{p}}{True_{p}+False_{N}}$$

(13) $$Sep=\frac{True_{N}}{False_{P}+True_{N}}$$

(14) $$Acc=\frac{True_{p}+True_{N}}{True_{p}+False_{p}+True_{N}+False_{N}}$$

(15) $$JSI=\frac{True_{p}}{True_{p}+True_{N}+False_{N}}$$

(16) $$MCC=\frac{\left(True_{P}\times True_{N}\right)-\left(False_{P}\times False_{N}\right)}{\sqrt{\left(True_{P}+False_{P}\right)\times \left(True_{P}+False_{N}\right)\times \left(True_{N}+False_{P}\right)\times \left(True_{N}+False_{N}\right)}}$$

**Figure 9:**
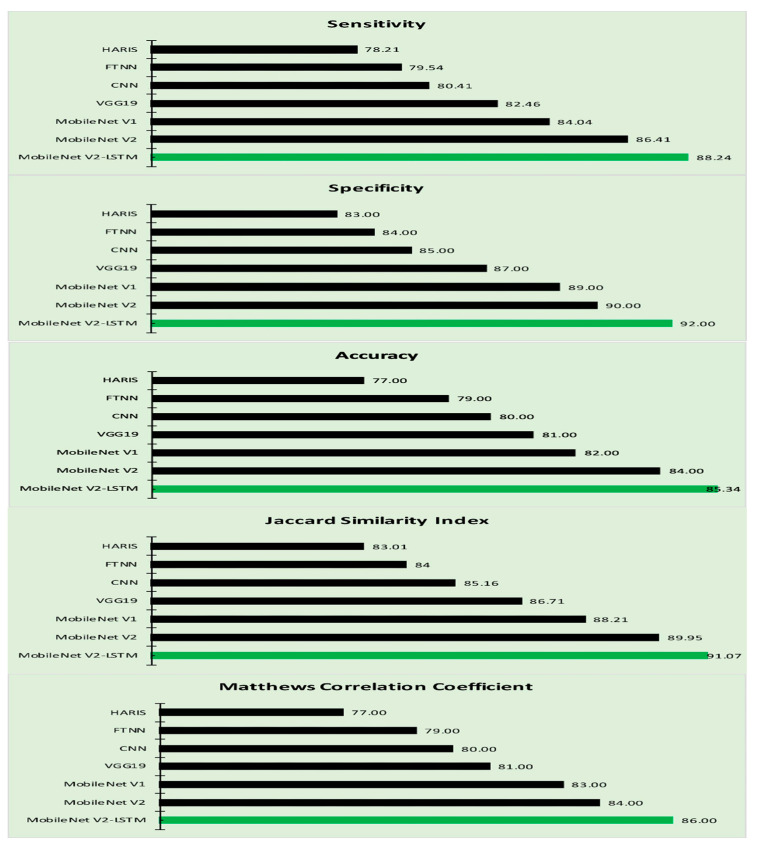
The performance of the MobileNet V2-LSTM model.

Table 3 reflects our proposed approach’s performance and other related approaches in terms of Sensitivity, Specificity, Accuracy, JSI, and MCC. The MobileNet-based models exhibited a better performance in classifying the region of interest with minimal computational efforts; the MobileNet V2 exhibited an optimal efficiency in disease classification [70]. The MobileNet V2 model encompassed LSTM which has an impact on the crucial parameters like learning rates and input and output gates that yield a better outcome. Plotting the results of Table 3 in Figure 9, it is visible that the proposed MobileNet V2-LSTM approach outperformed other state-of-the-art models in almost all performance sectors.

**Figure 10:**
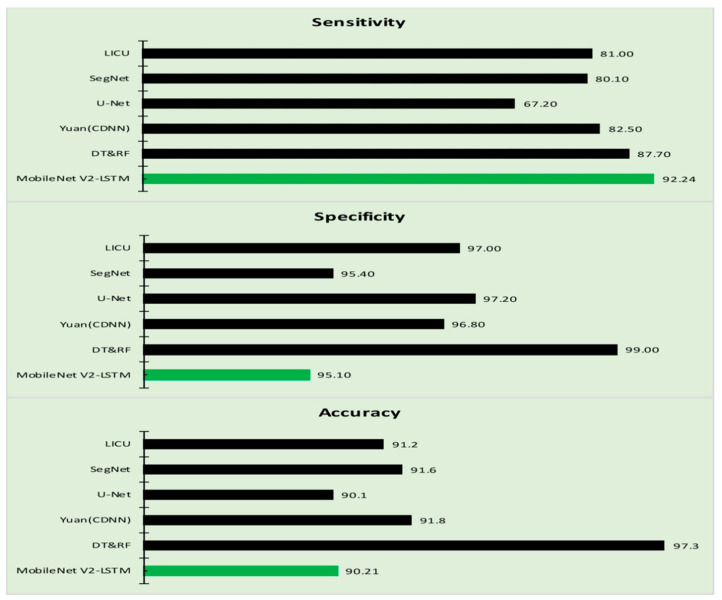
The comparative analysis of MobileNet V2-LSTM model.

The performance of the proposed model is compared against the various other approaches concerning the parameters like Accuracy, Sensitivity, and Specificity of each of the approaches like Decision Tree and Random Forest approaches, Lesion Index Calculation Unit (LICU) approach, Fuzzy Support Vector Machine with probabilistic boosting the segmentation, Compact Deep Neural Network, SegNet model, U-Net model, respectively [81,82,83,84,85], considered for comparative analysis that determine the efficiency of the model. Figure 10 is the graph that is obtained from the values of Table 4.

The proposed model outperformed compared to the various existing approaches. All the approaches are examined against the five classes of skin diseases. The proposed model is implemented against seven skin diseases classes as evaluations presented in Table 1 and Table 2. The proposed model’s performance has been observed as a steep incline in performance, reducing the number of classes for comparison. The other significance of the proposed model is that the computation efforts needed for the classification of the skin disease are comparatively low compared to the rest of the methods considered for evaluation. Experimentation is performed further to assess the progress of the skin disease through texture-based information [24,86]. Table 5 presents the progress of the disease through the metrics like Disease Core (DC) that represents the actual region of the tumor, and the Enhanced Disease (ED) is the region that has recently been affected by the disease that is approximated through the texture of the sin around the disease code and the entire region of the disease code and the enhanced disease is considered as the Whole Disease (WD). The experimental study is efficient in assessing the impact of the treatment on the disease. The progress in disease is likely to be more accurate when examined against the ground facts, and it would help take up the most suitable medication for controlling the disease. The confidence of obtained outcome is assessed through Equation (17):

(17) $$conf\left(d1\to d2\right)=\frac{support\left(d1\cap d2\right)}{support\left(d1\right)}$$

**Figure 11:**
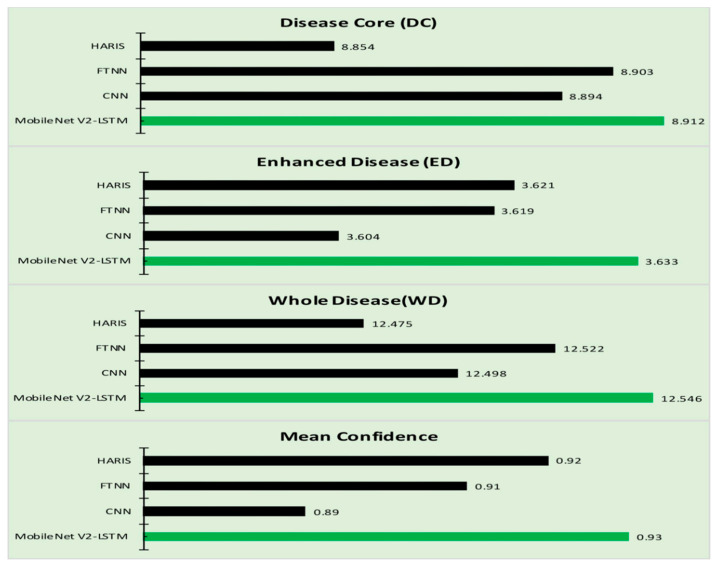
The progress of the disease growth.

The confidence mean in Table 5 is the value obtained on evaluating the mean of the confidence values observed on repeated experimentation. The robustness of the proposed approach can be determined from the mean of the confidence value that is assessed. The values after the decimal digits represent the deviation of the approximated from the ground facts. The values for the proposed approach are almost negligible compared to the other methods compared in the paper. Figure 11 represents the graphs obtained from Table 5, illustrating the disease growth progress that would support better treatment for the patients. The model is efficient in assessing the progress of diseased growth. The confidence value determines the average confidence level at which it determines the enhanced region of the disease. The proposed model is efficient in approximating the class of the disease more precisely with minimal computational efforts.

The incorporation of the LSTM component has enhanced the accuracy of the proposed approach. It can be observed from Table 3 that the proposed MobileNet V2 with LSTM model has outperformed over the other approaches like the HARIS, FTNN, CNN, VGG19, and conventional MobileNet V1, MobileNet V2 models in terms of Sensitivity, Specificity, Accuracy metrics alongside the MCC and JSI [87,88,89]. It can be analyzed that the proposed model is better than LICU, SegNet, U-Net, Yuan in terms of Sensitivity, Specificity, and Accuracy, as presented in Table 4.

**Figure 12:**
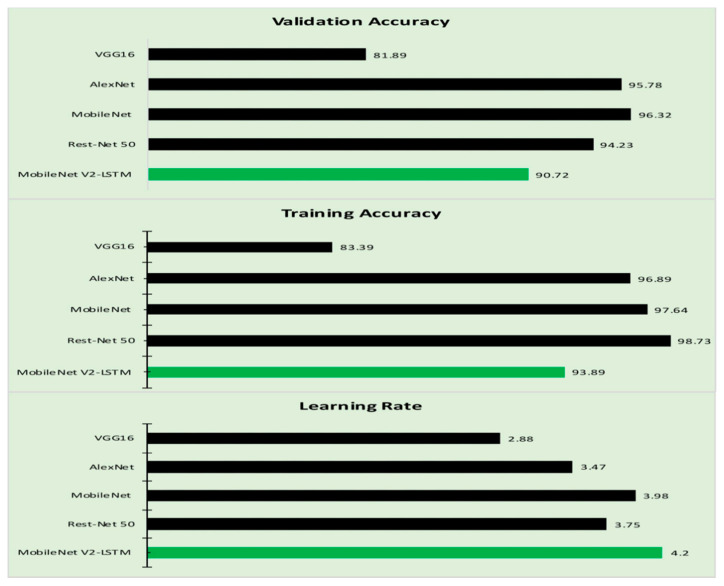
The hyperparameters of the proposed model.

Training loss and validation loss are two significant hyper-parameters that determine the preciseness of the proposed model. The training accuracy and the validation accuracy of the proposed model are evaluated against the similar parameters of other models considered in this study. Table 6 presents the Training and Validation accuracy of the various approaches [87,88,89,90,91]. Figure 12 represents the graphical representation of the values obtained from Table 6.

### 4.3. Execution Time

**Figure 13:**
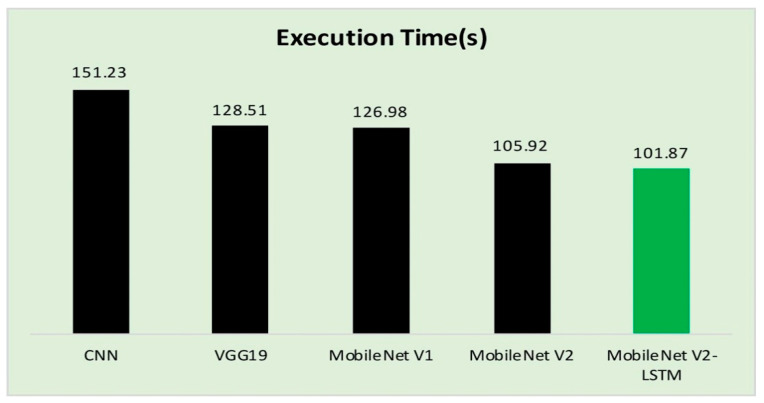
The execution time of MobileNet V2 with LSTM and other approaches.

In the process of evaluating the performance of the proposed model, the execution time of the validation phase is presented in Table 7, and Figure 13 in accordance to the existing studies. The proposed model consumed approximately around 1134 seconds for training the model over 20 epochs. The computational time to MobileNet V2 with LSTM over MobileNet V2 has not drastically reduced [92,93]. Still, MobileNet V2 exhibited a better prediction accuracy in terms of other performance evolution measures like Sensitivity, Specificity, and Accuracy.

The computational time of the proposed MobileNet V2 with LSTM is reasonably good, as shown in Table 7 which makes it feasible to incorporate the technology to run over the computationally lite weighted devices. Incorporating the LSTM module will assist in faster convergence by remembering the significant features necessary for the more rapid and accurate classification of the lesion images.

### 4.4. Practical Implications

**Figure 14:**
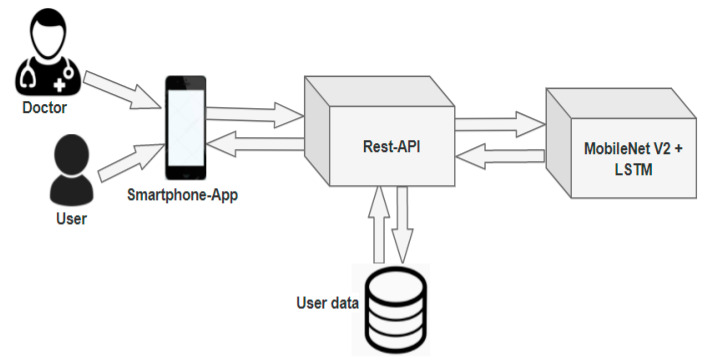
The framework of the proposed mobile application modules doctor, user, Rest-API for database connectivity, and the database.

The proposed model based on MobileNet V2 with LSTM is associated with the mobile application for ease of use for the patients/doctors to classify diseases based on the image fed as the input shown in one such application [94]. Figure 14 represents the architecture of the proposed model. The mobile app is designed to acquire the affected region’s image and the representational state transfer (Rest) API for securely storing the data in a remote server. NoSQL MongoDB is used in handling massive user-related data.

The proposed model is quite helpful for both the patients and the doctor in classifying the type of skin disease. The image captured using the mobile device is fed as the input for the interface. The interface then uses the MobileNet V2 with LSTM for processing the data. The MobileNet V2 can be implemented in an iOS platform through *netscope* and *netron* architecture. The information can either be transferred through the XML/JSON, or the model can be implemented in an iOS platform without separate space for the model. A flask framework can be used in web/mobile-based data access with a set of available libraries. The LSTM can be imported from the Keras libraries that are available for incorporating into the model; the integration of the LSTM is almost the same as the Recurrent Neural Network architecture.

**Figure 15:**
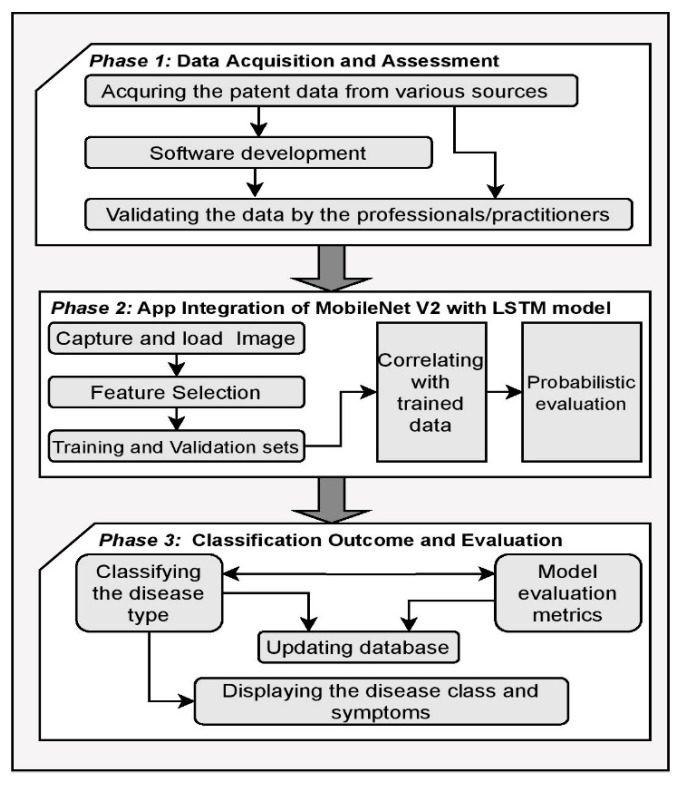
The mobile framework on incorporating MobileNet V2 with LSTM.

The proposed framework for the practical implication involves multiple phases in the process of classifying the type of the skin disease presented through Figure 15. In the initial phase, the data are acquired and assessed by the professionals and practitioners for the type of disease for accurate training of the model. The second phase of the framework concerns the app integration of the proposed MobileNet V2 with LSTM model. In this phase, the image of the affected region is captured and fed as the input for the model, the features of the input image are identified for correlating the features with the trained data for predictions. The probabilities of the particular type of diseases are approximated in this phase to determine the class of the disease. In the third phase, the classification outcome and the evaluation of the model are performed. The disease classification probability determines the class of the disease, and the outcome of the predictions are evaluated against the various evaluation metrics and the information is updated in the database for the feature perception [95].

**Figure 16:**
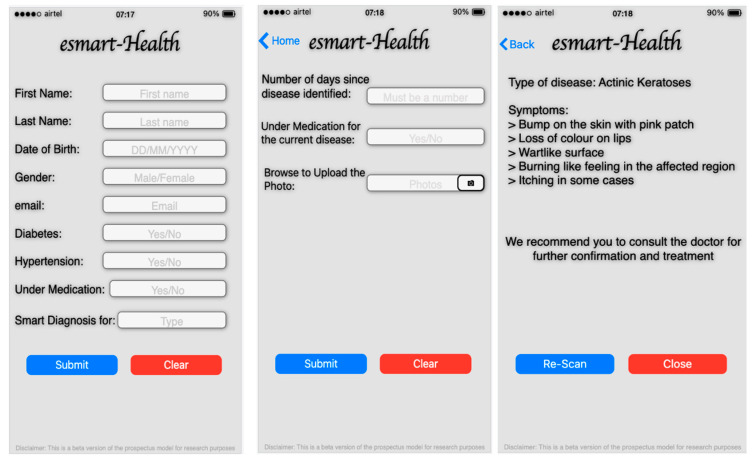
The interface of the mobile application to gather user’s data and prediction result interface of mobile application.

Figure 16 represents the screens acquired from the prospectus model, the user’s information that includes the name, date of birth, gender, email, and the date related to the current health conditions like diabetes, hypertension, etc., entered by the user. The type of diseases is selected on the home page, which redirects users to the appropriate page where the user has the provision to upload the image of the affected region as showing the second image of Figure 14 and the data like the number of days since effected. Upon recognizing the suitable type of skin disease, it will be returning the disease’s details and the symptoms associated with the disease, as shown in Figure 14. The details provided will help the physician, radiologist, and the patient in the preliminary assessment of the disease.

The performance of the proposed Mobilenet V2 with LSTM is evaluated through various assessment metrics, and the implemented results are presented along with the graphs of the hyperparameters. It is evident from the obtained results that the proposed model’s performance for lesion classification is reasonably fair with minimal computational time than the other approaches. The proposed model needs a considerable lesser computational effort in performing the classification of the images, which makes it suitable to deploy in mobility devices. The prospectus application that works with the proposed model can precisely identify the skin disease for the image that is captured.

## 5. Conclusions

The proposed model based on the MobileNet V2 and LSTM approach proved efficient for skin disease classification and detection with minimal computational power and effort. The outcome is promising, with an accuracy of 85.34% when experimented with and compared with other methods over the real-time images acquired from Kaggle [11]. The MobileNet V2 architecture is designed to work with a portable device with a stride2 mechanism. The model is computationally effective, and the use of the LSTM module with the MobileNet V2 would enhance the prediction accuracy by maintaining the previous timestamp data. The information related to the current state through weights optimizations would make the model robust. It is also compared against various other conventional models like CNN, FTNN, and HARIS. It is observed that the proposed model has outperformed in classification and analyzing the progress of the tumor growth based on the textured-based information as presented in the Results and Discussion section. The bidirectional LSTM may further improvise the performance of the model. In the practical implementation of the proposed model, an association of the front end designed through the android studio/SSDLite/DeepLabv3+ and the business model built over Kaggle has taken tremendous efforts in integrating either of the models. However, at the present point, there is a range of shortcomings that must be resolved in future work. The model’s precision is dramatically decreased to just below 80 percent when checked on a series of photographs captured in poor illumination conditions distinct from those used during testing. Eventually, the proposed approach is not designed to replace but rather to supplement existing disease-diagnostic solutions. Laboratory test results are always more trustworthy than diagnoses based solely on visual symptoms, and visual inspection alone often challenges early diagnosis.

## 6. Future Works

The proposed model is computationally efficient as it is designed to work on top of lightweight capability devices. The proposed MobileNet V2 with the LSTM model needs a more significant number of parameters for better accuracy. The considered input image and the MobileNet V2 with LSTM model’s resultant outputs have no significant randomness to explore all possible patterns in the assessment process. Alongside the bottleneck in residual connections in the proposed architecture, the model yields higher accuracy with minimal effort. The model can be further improved by incorporating the self-learning capability and knowledge acquisition from its previous experiences. The efforts on training the model can be considerably reduced. However, the model must be mechanized to assess the impact of features extracted for each strategy, and the incorporation of randomizing components is necessary. The researchers recommend that future research be performed to examine the feature extraction actions based on biomarkers, even though there is ample data, depending on the specific findings. Biomarkers effectively identify the disease from the supplementary data like the genomic, protein sequences, and pathological data in addition to the imaging data. It is recommended to consider lightweight security when transmitting physiological and biological data in health networks, and a user-friendly smart device app, which can display alarms and communicate between patients and physicians in eHealth and telehealth environment to securely exchange and transmit data [96,97].

## Figures and Tables

**Table 1:** The related work of the machine and deep learning approaches for image classification.

Reference	Approach	Objective	Challenges of the Approach
[22]	Morphological Operations	Morphological operations involve the dilation and erosion that are efficient in identifying the image features that help determine the abnormality. It works through the structuring element.	Identifying the optimal threshold is crucial and not suitable for analyzing the disease region’s growth through morphology operations. The process of applying the structuring elements for the skin disease classification does not yield an accurate result.
[48]	K-Nearest Neighborhood	KNN based model works without the training data in classifying the data through the feature selection and similarity matching for categorizing the data. It works through the distance measure as the mode of identifying the correlation among the selected features.	KNN-based classification model, the accuracy of the outcome is directly dependent on the quality of underlying data. Additionally, in the case of a larger sample size, the prediction time might be significantly high. The KNN model is subtle to the inappropriate features in the data.
[20,24,59]	Genetic Algorithm	The genetic algorithm relies more on a probabilistic approach by randomly selecting the initial population. It performs the crossover and the mutation operations simultaneously until it reaches a suitable number of segments.	The Genetic Algorithm does not guarantee the global best solution and too much time to converge.
[28,60]	Support Vector Machine	Support Vector Machine is efficient in handling the high dimensional data with minimal memory consumption.	Support Vector Machine approach is not appropriate for noisy image data and identifying the feature-based parameters is a challenging task.
[31,35]	Artificial Neural Networks	Artificial Neural Networks are efficient in recognition non-linear associations among the dependent and independent parameters by storing the data across the network nodes.	Artificial Neural Network models are efficient in handling the contexts like inadequate understanding of the problem. However, the approach there is a chance of missing the image’s spatial features, and diminishing and exploding the gradient is a significant concern.
[32,34]	Convolutional Neural Networks	Convolutional Neural Network models are efficient in the automatic selection of the essential features. The CNN model stores the network nodes’ training data as multi-layer perceptrons rather than storing it in the auxiliary memory.	CNN approach fails to interpret the object’s magnitude and size. Additionally, the model needs tremendous training for a reasonable outcome, apart from the challenge like the spatial invariance among the pixel data.
[61]	Fully Convolutional Residual Network	Fully Convolutional Residual Network uses the encoder and decoder layers that utilize high-level and low-level features to classify the objects from the image.	The Fully Convolutional Residual Network is efficient in handling the overfitting issue and the degradation problem. However, the model is complex in design and real-time execution. In addition, adding the batch normalization would result in making the architecture more intricate.
[36]	Fine-tuned Neural Networks	Fine-Tune Neural Network is efficient in handling the novel problem with pre-trained data through inception and update stages.	In FTNN approach, when the elements are fed with new weights, it forgets the previously associated weight that may impact the outcome.
[41,42]	Gray Level Co-occurrence Matrix (GLCM)	Gray Level Co-occurrence Matrix (GLCM) is a statistical approach that performs the object’s classification by analyzing spatial association among the pixels based on the pixel texture.	The GLCM approach needs considerable computational efforts, and characteristics are not invariant with rotation and texture changes.
[43,44]	Bayesian classification	The Bayesian classification-based approach efficiently handles discrete and continuous data by ignoring the inappropriate features for both the binary and multi-class classifications.	The Bayesian Classifier is not suitable for handling the unsupervised data classification, fails in independent predictors, and is widely known as an inappropriate probabilistic model.
[45,46]	Decision Tree	Decision Tree-based models are used in handling both the stable and discrete data that performs the prediction through a rule-based approach. It is proven to be productive in managing non-linear parameters.	In Decision Tree models, a small change in the input data would result in an exponential growth in the outcome makes the model unstable. Overfitting is the other issue associated with the decision tree-based models.
[50,51,52]	Ensemble models	Ensemble models are proven to be better prediction models with a combination of various robust algorithms. They are efficient in analyzing both the linear and complex data patterns by combining two or more complex models.	Ensemble models do have the overfitting issue, and the ensemble model fails to work with unknown discrepancies. The model minimizes the understandability of the approach.
[53,54]	Deep Neural Networks	Deep Neural Networks-based models can work with structured and unstructured data. The models can still be able to work with unlabeled data and can yield a better outcome.	The models like the Inception V3 model [62,63] is used in classifying skin disease. On experimentation, the authors have found the model is not suitable for the disease with multiple lesions.

**Table 2:** The configuration information of the proposed model.

Implementation Configuration Parameters
Model: Torch Vision, Mobilenet-V2
Base learning rate: 0.1
Learning rate policy: Step-Wise (Reduced by a factor of 10 every 30/3 epochs)
Momentum: 0.95
Weight decay: 0.0001
Cycle Length: 10
PCT-Start: 0.9
Batch size: 50

**Table 3:** The performance metrics of the various approaches.

Algorithms	Sensitivity (%)	Specificity (%)	Accuracy (%)	JSI (%)	MCC (%)
HARIS [25]	78.21	83.00	77.00	83.01	77.00
FTNN [77]	79.54	84.00	79.00	84.00	79.00
CNN [32]	80.41	85.00	80.00	85.16	80.00
VGG19 [78]	82.46	87.00	81.00	86.71	81.00
MobileNet V1 [71]	84.04	89.00	82.00	88.21	83.00
MobileNet V2 [80]	86.41	90.00	84.00	89.95	84.00
MobileNet V2-LSTM	88.24	92.00	85.34	91.07	86.00

**Table 4:** The performances of the various algorithms.

Algorithm	Sensitivity (%)	Specificity (%)	Accuracy (%)
LICU [81]	81.0	97.0	91.2
SegNet [58]	80.1	95.4	91.6
U-Net [60]	67.2	97.2	90.1
Yuan (CDNN) [81]	82.5	96.8	91.8
DT&RF [81]	87.7	99.0	97.3
MobileNet V2-LSTM	92.24	95.1	90.21

**Table 5:** The progress of the disease growth.

Algorithm	Disease Core (DC)	Whole Disease Area (WD)	Enhanced DISEASE (ED)	Confidence (Mean Value)
HARIS [25]	8.854	12.475	3.621	0.92
FTNN [77]	8.903	12.522	3.619	0.91
CNN [32]	8.894	12.498	3.604	0.89
MobileNet V2-LSTM	8.912	12.546	3.633	0.93

**Table 6:** The training, validation accuracy, and learning rate.

Algorithm	Training Accuracy (%)	Validation Accuracy (%)	Learning Rate (%)
VGG16 [65]	83.39	81.89	2.88
AlexNet [65]	96.89	95.78	3.47
MobileNet [80]	97.64	96.32	3.98
Rest-Net 50 [65]	98.73	94.23	3.75
MobileNet V2-LSTM	93.89	90.72	4.20

**Table 7:** Execution Time.

Algorithm	Execution Time(s)
CNN [32]	151.23
VGG19 [78]	128.51
MobileNet V1 [71]	126.98
MobileNet V2 [80]	105.92
MobileNet V2-LSTM	101.87

## Data Availability

The data HAM10000 from Kaggle is considered for the experimental study in the paper. The data set consists of 10,000 dermatoscopic images of various individuals worldwide, with the divergent type of skin diseases. The data is openly available from the link https://kaggle.com/kmader/skin-cancer-mnist-ham10000 (accessed on 17 April 2021).
